# New Digital Metal-Oxide (MOx) Sensor Platform

**DOI:** 10.3390/s18041052

**Published:** 2018-03-31

**Authors:** Daniel Rüffer, Felix Hoehne, Johannes Bühler

**Affiliations:** Sensirion AG, CH-8712 Stäfa, Switzerland; felix.hoehne@sensirion.com (F.H.); johannes.buehler@sensirion.com (J.B.)

**Keywords:** metal oxide, gas sensor, industrialization, miniaturization, microelectromechanical systems (MEMS), micro-heater, indoor air quality

## Abstract

The application of metal oxide gas sensors in Internet of Things (IoT) devices and mobile platforms like wearables and mobile phones offers new opportunities for sensing applications. Metal-oxide (MOx) sensors are promising candidates for such applications, thanks to the scientific progresses achieved in recent years. For the widespread application of MOx sensors, viable commercial offerings are required. In this publication, the authors show that with the new Sensirion Gas Platform (SGP) a milestone in the commercial application of MOx technology has been reached. The architecture of the new platform and its performance in selected applications are presented.

## 1. Introduction

We are in the midst of the so-called sensor revolution: the Internet of Things (IoT), comprising smart appliances and smart gadgets, as well as ever more powerful wearable and mobile consumer devices fuel the demand for smarter sensor solutions. In particular, the field of environmental sensing has received growing interest, mostly due to rising awareness of the environmental effects on health and human well-being. Air pollution has been universally acknowledged as a significant cause of health problems and even premature death [1,2,3,4,5]. As a consequence, there is a growing demand for personal and affordable air-quality monitoring solutions.

Until now, commercially available metal-oxide (MOx) sensors had technical shortcomings which limited their usage in many interesting applications.

Even though the selectivity of MOx sensors can be tuned and notably improved, long-term stability in the presence of siloxanes will deteriorate their accuracy over time or even make it impossible to use sensors in applications with high siloxane loads such as mobile phones [6,7,8]. Moreover, most available sensors do only offer a simple analog interface. The output signal is provided in form of a resistance for which additional circuitry has to be implemented. On top of this, a calibration routine has to be realized if calibrated concentration readings are required. Because the heating elements are mostly operated in an open loop, such analog sensors are often susceptible to changes in environmental conditions. Additionally, many of the available solutions are not suited for mobile applications due to their size and prohibitive power consumption. Furthermore, while the broadband sensitivity of MOx sensors is beneficial for the detection of a wide variety of volatile organic compounds (VOC) in indoor air-quality (IAQ) monitoring, it is a limitation in applications which require a certain level of selectivity to specific gases. Among the different approaches to improve selectivity [6,9,10,11,12,13,14,15,16,17,18,19,20,21], temperature modulation [8,10,22,23] and combination of multiple sensing elements with different intrinsic selectivity [8,10] are promising approaches to improve the selectivity of MOx sensors.

While there are sensors on the market which fulfill individual aspects of these requirements, there are, to the knowledge of the authors, no commercially available products which conform to all of them. For instance, as of today, the most prominent commercially available sensors in the market are analog [24,25,26]. In order to tackle the unique challenges for gas sensing in IoT and mobile applications, Sensirion developed the Sensirion Gas Platform (SGP) multi-pixel gas sensor. Multiple sensing elements are combined with a siloxane resistance, which is unprecedented for a commercially available MOx sensor. The sensing elements are integrated with a digital interface into a small surface mount device (SMD). In combination with the on-chip calibration data, the digital interface greatly simplifies the integration of the SGP into different applications, since the output signal can be directly used by customers for air-quality indication without further processing.

## 2. Materials and Methods

### 2.1. Sensor Architecture

**Figure 1:**
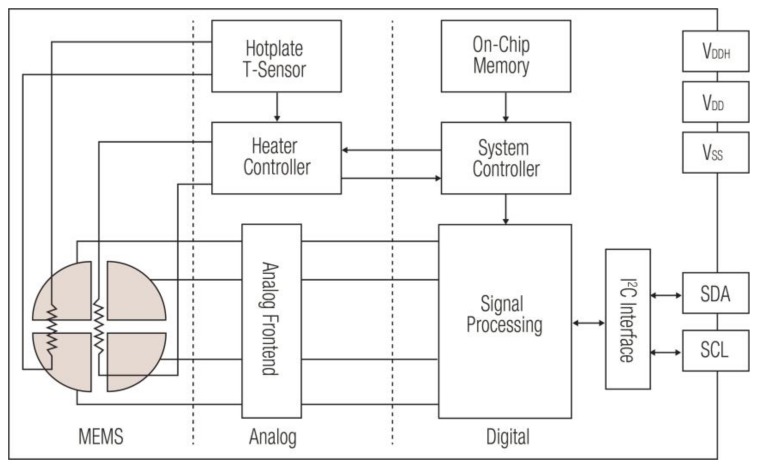
Block diagram of the SGP multi-pixel gas sensor platform [27], including the I^2^C (inter-integrated circuit) bus interface, consisting of serial data (SDA), serial clock (SCL), supply and ground voltages V_DD_ and V_SS_. V_DD_ and the hotplate supply voltage V_DDH_ can be shorted.

**Figure 2:**
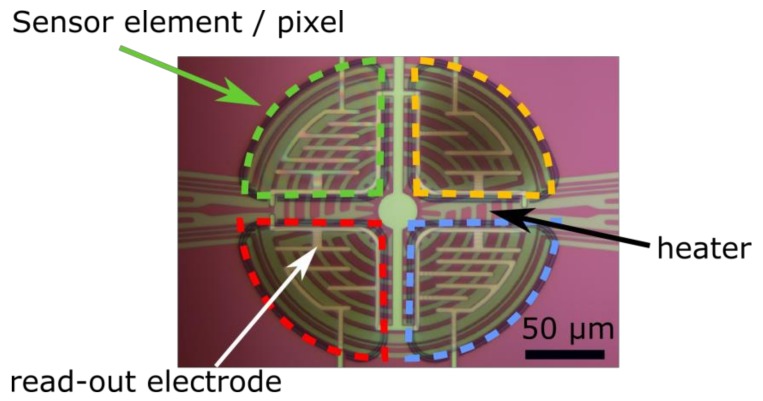
Micrograph showing the four sensing elements (indicated by colored shapes), the read-out electrodes and the heater element.

The SGP offers a complete gas sensor system integrated into a very small 2.45 × 2.45 × 0.9 mm^3^ dual flat no-lead (DFN) surface mount package. Sensirion’s proprietary CMOSens^®^ technology (Sensirion AG, Stäfa, Switzerland), allows the co-integration of analog and digital electronics together with a micro-hotplate and the sensing elements on a single chip, as shown in the block diagram in Figure 1. Four MOx sensing elements based on layers of MOx nanoparticles are deposited on a micro-hotplate (Figure 2). Each sensing element can be measured separately by read-out electrodes. To guarantee stable operation independent of the surrounding temperature, a heater and a temperature sensor are integrated on the hotplate to actively control its operating temperature.

The signals from the four sensor elements are measured by an optimized amplifier covering a measurement range of eight orders of magnitude. This is crucial for measuring a wide variety of different MOx sensing materials as well as different gases and gas concentrations with a single hardware platform. The signals are further processed in the digital signal-processing stage with algorithms e.g., for averaging, baseline compensation and humidity compensation. In addition, individual calibration parameters are written during production into an on-chip one-time programmable memory. This allows conversion of the sensor raw signals into calibrated output signals such as those for concentrations of volatile organic compounds.

### 2.2. Manufacturing Process

**Figure 3:**
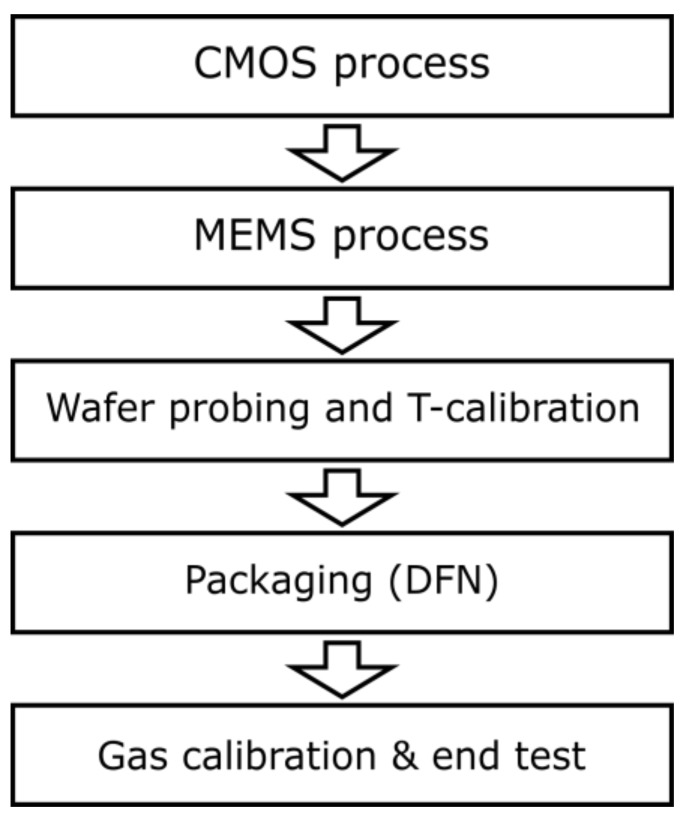
Process flow.

Sensirion sensors are fabricated with a high level of automation and quality control. Figure 3 gives an overview of the manufacturing process of an SGP gas sensor. The structures for the logic elements of the chip are produced in a Bipolar-CMOS-DMOS (BCD) technology process, where CMOS and DMOS. In a subsequent MEMS (microelectromechanical systems) step the heater, temperature sensor and the MOx read-out electrodes are deposited and patterned. A backside cavity is etched via deep reactive-ion etching (DRIE), followed by the MOx deposition. The currently available products, SGPC3 and SGP30 (Sensirion AG, Stäfa, Switzerland), are based on SnO_2_ materials with palladium doping between 0.1% and 5%. In future products, further material combinations can be integrated to take full advantage of the multi-pixel platform. After wafer probing, the individual hotplate temperature sensors are calibrated on the wafer level. Following leadframe assembly, dicing and packaging, each sensor chip is calibrated to the target gases. In the case of indoor air-quality monitoring, ethanol and hydrogen (H_2_) are used as calibration gases (for more details about the gas choice, please refer to Section 3). After calibration, a final end-test and outgoing inspection of each chip is performed before packaging the chips into reels.

The entire process is tightly monitored at every step and strict grading ensures that only sensors which adhere to the specifications and can be readily used by the customer are shipped.

### 2.3. Experimental Methods

The sensors were reflow soldered on either custom printed circuit boards (PCBs) or flexible PCBs. To increase sample size, the solid PCBs allow up to 32 sensors to be accommodated per board. Because the sensor chips contain all the necessary analog circuitry, the read-out electronics have been kept simple. Apart from a stable 1.8V power supply, only an i^2^c bridge and, in the case of the multi-sensors boards, an i^2^c multiplexer chip, is required. The entire electronics were designed in-house.

For the field measurements, the read-out electronics were connected to a miniature computer (Raspberry Pi 2, Raspberry Pi Foundation, Cambridge, UK) which controlled the data-acquisition and the data upload via WiFi. As a reference for CO_2_ a non-dispersive infrared (NDIR) sensor (Senseair K30, Senseair AB, Delsbo, Sweden) was connected. The devices were placed in regular meeting rooms and were operated without interruption.

In the laboratory measurements, gas mixing systems (GMS) with up to 8 channels were employed. Each channel was controlled with a Sensirion SFC5400 mass flow controller (MFC) (Sensirion AG, Stäfa, Switzerland). For the dry channel, air generated by a zero-air generator was used. In the wet channel, the zero air was passed through a water bubbler. To avoid errors due to incomplete saturation of the air in the bubbler, the ratio of dry and wet channel was controlled in a closed loop using a Sensirion SHT temperature and humidity sensor (Sensirion AG, Stäfa, Switzerland). To stabilize the temperature, the bubbler and the measurement chamber were installed in environmental chambers manufactured by Thunder Scientific (Thunder Scientific Corp., Albuquerque, NM, USA). The temperature uniformity and stability is specified to ±0.1 °C. The gas provided by the MFCs was fed through a metal tube acting as a heat-exchanger. The total flow was chosen to be 0.5 L/min. To ensure a leak-free system, the total flow was monitored with a Sensirion SFM5400 mass flow meter (MFM) (Sensirion AG, Stäfa, Switzerland) after the measurement chamber before the gas was fed to the exhaust.

To simulate stress under siloxane load, a separate GMS was employed to avoid contamination. The system was equipped with an additional bubbler containing decamethylcyclopentasiloxane (D5). The bubbler is circulated by water and the temperature is actively regulated using a chiller to yield a concentration of about 500 ppm [28]. Subsequently, humid air is mixed in to yield 250 ppm D5 at 50% relative humidity.

The sensors from other manufacturers were operated as specified in their respective datasheets. To supply the sensors and measure their resistance *R*, laboratory grade benchtop power supplies and multimeters (Agilent E3647A and Keysight 34461A, Keysight Technologies, Santa Rosa, CA, USA) were employed. Prior to their exposure to D5 or their operation in the phone, the sensors were calibrated to ethanol in the GMS using ethanol steps ranging from 0.3–30 ppm. Then, the usual power law:

(1) $$c_{EtOH}\left(R\right)=c_{0}{\left(\frac{R}{R_{0}}\right)}^{\frac{1}{n}}$$

is fitted to obtain a calibration unique to each sensor. $R_{0}$ is the baseline resistance. The sensitivity *n* and the scaling factor $c_{0}$ are fitting parameters.

## 3. Results

### 3.1. Siloxane Stability

**Figure 4:**
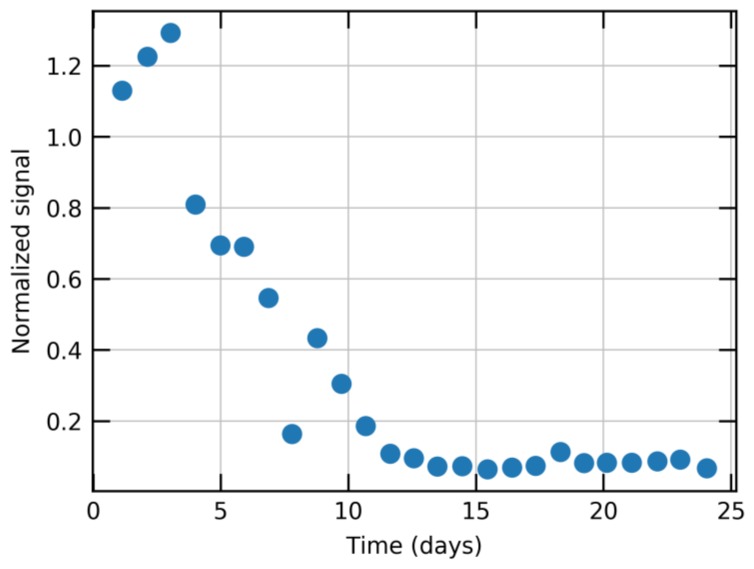
Normalized signal of a traditional metal-oxide (MOx) sensor to 0.5 ppm ethanol as function of operating time. The sensor was mounted inside a mobile phone. After 2 weeks, the sensitivity to ethanol is close to zero.

In most applications in consumer and appliance markets, stable and maintenance-free operation of sensors over a device lifetime of 3–10 years is required. On the other hand, MOx-based gas sensors suffer from poor long-term stability when they are operated in atmospheres containing even very low concentrations of siloxanes [6,7,8]. The degradation caused by siloxanes typically translates into a significant decrease in VOC sensitivity and a strong increase of response time, potentially caused by silicon dioxide (SiO_2_) formation [6,29]. Unfortunately, silicon-containing compounds are found in many products used in everyday life such as cosmetics, cleaning agents or plastic parts [30,31,32,33] and, therefore, siloxanes are present in all relevant operational environments. In fact, recent studies have shown that siloxanes are the most abundant volatile organic compound (VOC) emitted by humans due to their extensive use in personal care products such as antiperspirants [34]. The overall siloxane background concentration determines the time scale of degradation and, therefore, the total sensor lifetime. This problem is particularly pronounced in applications like mobile phones, where the sensor is constantly exposed to high siloxane concentrations originating from various parts of the mobile phone. Figure 4 shows data recorded with a commercially available MOx sensor which was mounted and operated inside a mobile phone in an ambient indoor environment. At regular intervals the phone with sensor was exposed to 0.5 ppm of ethanol. For this sensor, like for most air-quality sensors, this amount of ethanol corresponds to a typical air-quality signal. After an initial increase, the signal drops significantly within a few days. After only 2 weeks, the sensitivity to ethanol has decreased to almost zero.

The core technology of the SGP, MOXSens^®^ (Sensirion AG, Stäfa, Switzerland), provides the sensor with a unique robustness against contamination by siloxanes. This is achieved by a combination and optimization of the sensing material, operation mode, and the on-chip evaluation of multiple signals from different sensing elements. To simulate the effect of siloxane exposure during multiple years of operation, an accelerated lifetime test can be employed. For this, the sensors are characterized in a GMS with clean zero air and a fixed concentration of a target gas, in this case ethanol at 10 ppm. Subsequently, the sensors are operated in an atmosphere containing an elevated concentration of 250 ppm of D5 for up to 200 h. D5 is chosen as it is the most abundant siloxane in most environments and commercial products [30,31,32,33]. Concentration and exposure time are chosen to approximate 10 years of operation in a background of 40 ppb of siloxanes [35]. After exposure, the sensors are re-characterized using the same ethanol concentration of 10 ppm.

**Figure 5:**
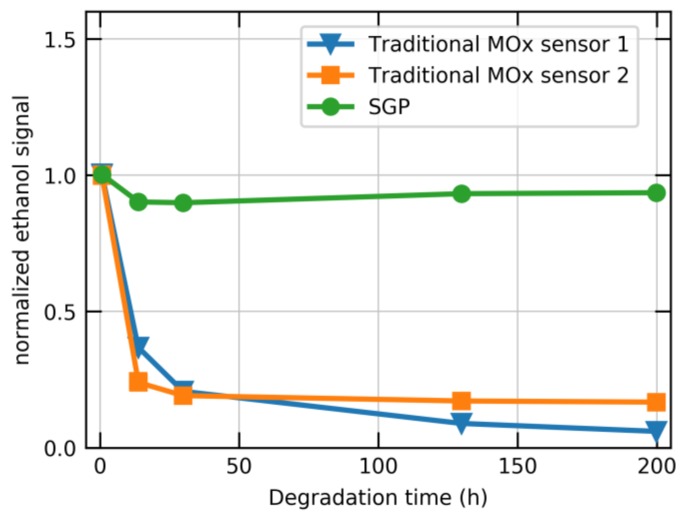
Long-term stability in an accelerated life-time test. The normalized ethanol concentration for different degradation time intervals is plotted for two commercially available sensors (blue and orange) and one Sensirion SGP sensor (green). The sensors have been calibrated before the accelerated lifetime test. The signals are normalized to their respective values before the test.

In Figure 5, the signal response, normalized to the signal prior to D5 exposure, is shown as a function of degradation time. In addition to a Sensirion sensor, two commercially available MOx sensors for the IAQ market are shown. While the Sensirion sensor only shows a minor, initial signal variation, the traditional sensors exhibit a significant decrease in their sensitivity of more than 90% of the initial signal response. The enhanced siloxane resistance significantly improves the long-term stability and accuracy of the SGP.

### 3.2. Total Volatile Organic Compounds 

The widespread use of new products and building materials has resulted in increased concentrations of indoor pollutants, in particular VOCs. These VOCs originate from a number of different sources including building materials, cleaning agents, furniture and indoor chemical reactions [36,37]. A number of systematic human exposure studies have reported a decrease of cognitive abilities and various adverse health effects caused by exposure to elevated VOC levels [38,39]. As a practical time and cost-effective method of surveying indoor environments for contamination, the total VOC (tVOC) concept has been introduced [40]. The term tVOC refers to the total concentration of VOCs present simultaneously in the air. Global consensus has resulted in the emergence of guidelines for tVOC standards of indoor air quality issued by governmental organizations in different countries. The maximum tVOC levels that are considered as acceptable range from ~0.6 to ~1 mg/m^3^ [40].

**Figure 6:**
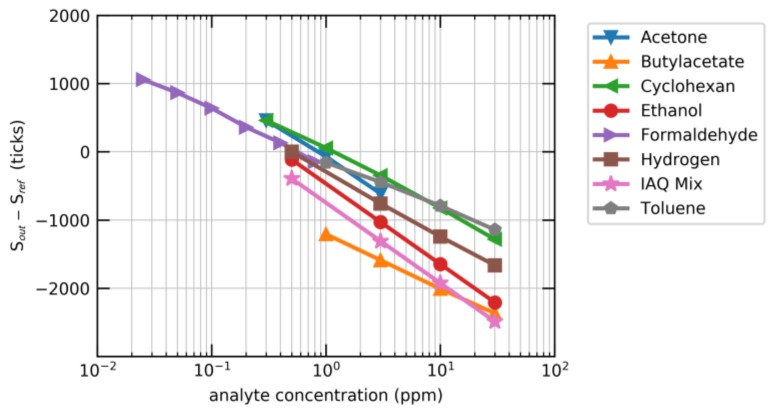
Sensor output (*S*_out_) normalized to the sensor signal at 0.5 ppm H_2_ (*S*_ref_) as function of the analyte concentration for various gases. Indoor air quality (IAQ) Mix refers to a gas mixture proposed by Mølhave et al. [39] representing a typical mixture of volatile organic compounds (VOCs) found in indoor environments.

Laboratory techniques to determine VOC concentrations, such as flame ionization detectors (FID) or gas chromatography–mass spectrometry (GC–MS), are either too expensive, too power-intensive or too large to be applicable in compact and affordable IAQ devices in e.g., smart home applications. For these applications, MOx sensors are the preferred technology due to their high sensitivity and broadband-sensing capabilities. This is illustrated in Figure 6 for Sensirion’s SGP30 sensor, where the sensor output is shown as a function of the gas concentration for different gases representing important classes of indoor air pollutants. The signal is calibrated such that the sensor output *S*_out_ follows:

(2) $$\ln\left(\frac{c}{c_{\mathrm{ref}}}\right)=\frac{S_{\mathrm{ref}}-S_{\mathrm{out}}}{a}$$

where *c* is the applied ethanol concentration, *S*_ref_ the value of *S*_out_ at a reference concentration *c*_ref,_ and *a* = 512 a scaling factor. In this graph, *S*_out_ is normalized to the sensor signal at 0.5 ppm H_2_, which corresponds to the atmospheric background concentration and thereby represents clean air [41]. The SGP shows high sensitivity to different VOCs, even in the sub-ppm concentration range which is most relevant for most indoor air-quality applications.

The different existing technologies, such as FID, photo-ionization detectors (PID) or MOx sensors, have varying sensitivities to each analyte [42,43]. These response curves are related to the employed technologies and do not reflect the relevance of the single gas components. Therefore, the use of gas mixtures with a defined but broad range of VOCs have been proposed to simulate IAQ levels [39,40,44,45]. To overcome impracticalities with such complex gas mixtures during factory calibration and testing, the sensor output is usually calibrated to a single gas. Which gas is suited best depends on the particular sensor technology. Ideally, the sensor should have a similar sensitivity to the single gas and a typical mixture of indoor VOCs.

**Figure 7:**
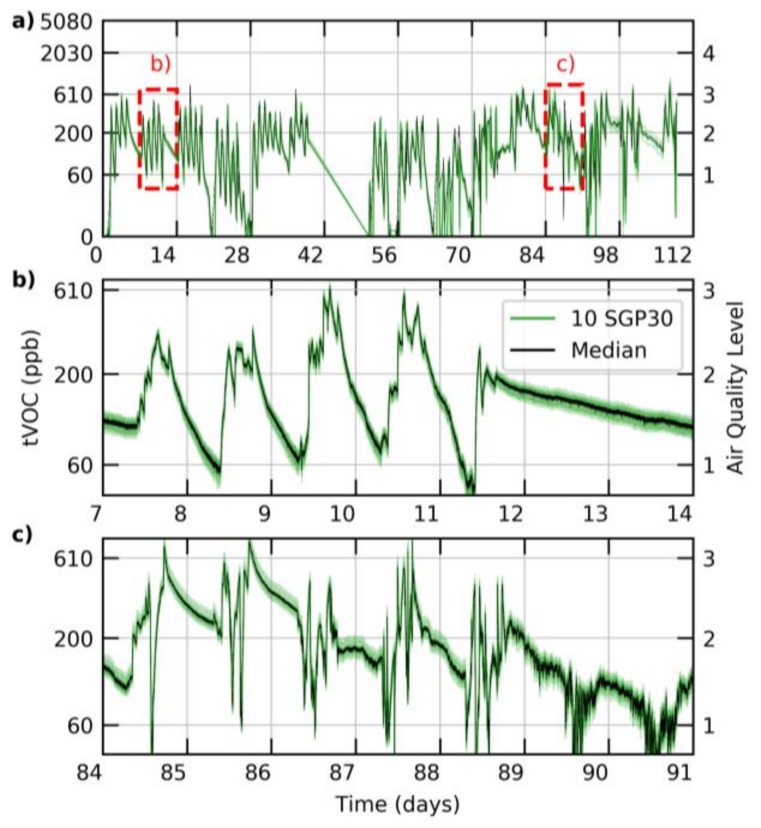
Total VOC (tVOC) signal in a typical meeting room, recorded with 10 different SGP sensors (green) over a period of about 4 months (**a**); the median is given as black line. The two red rectangles indicate the position of the zoom-ins provided in panels (**b**,**c**). On the secondary y-axis, the maximum values of the corresponding air-quality levels are indicated [46].

In the case of the SGP, identical sensitivities can be found for ethanol (red circles in Figure 6) and the gas mixture proposed by Mølhave et al. [39] (pink stars). The sensor can thus be calibrated to ethanol in the fabrication process. In the field, this raw ethanol signal is baseline-corrected and converted using the known conversion factor to yield an absolute tVOC output. All the computations are performed on-chip using the stored calibration parameters. Figure 7 depicts tVOC signals for 10 sensors which were operated in parallel for about 4 months in a meeting room. Colored spans indicate internationally accepted tVOC air quality levels [46]. The impact of meetings on the air quality can be monitored over the entire period simply by reading the sensor without the need for additional data processing or recalibration. In addition, the SGP production process and calibration ensure that the sensors show negligible device-to-device variation.

### 3.3. Humidity Cross-Sensitivity

It is well known that MOx sensors are sensitive to water vapor. In general, literature treats humidity as a reducing gas [47,48]. In reality, the picture can be more complex and the microscopic understanding is still the subject of extensive research [49,50]. From the application point of view, the cross-sensitivity to humidity is treated as nuisance and should be reduced as much as possible.

**Figure 8:**
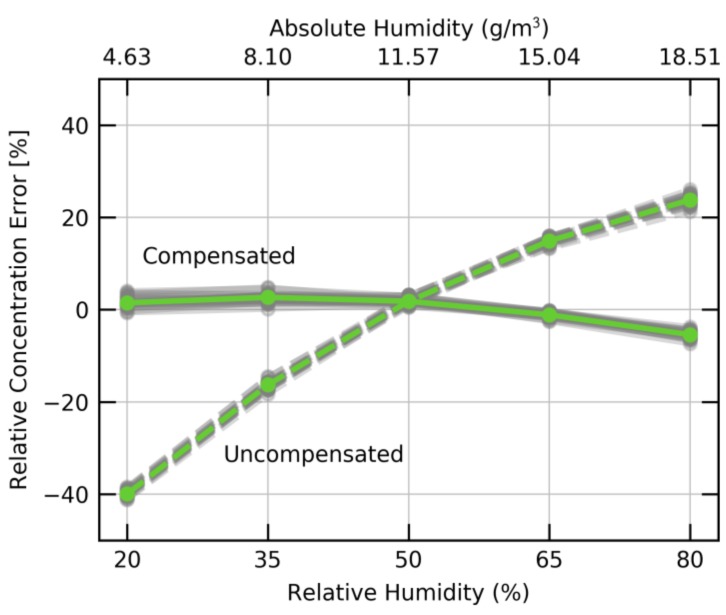
Relative error of the ethanol concentration (dotted line) at 10 ppm ethanol as function of humidity at 25 °C. Data of 32 SGP30 sensors (grey) and the median (green) is plotted. Additionally, the signal after on-chip correction using an external humidity signal is shown (solid line).

In Figure 8, the humidity dependence of the SGP30 at an ambient temperature of 25 °C and an ethanol concentration of 10 ppm is plotted. The graph shows the relative error in ethanol concentration for 32 SGP30 sensors. The experiment was performed in a temperature-stabilized GMS. The ethanol concentration output has a relative error of up to 40% depending on the humidity. However, the humidity dependence has very little variance over the entire set of sensors. As such a humidity dependency is undesired in most applications, the sensor offers an on-chip humidity compensation. Using the signal from an external humidity sensor like the Sensiron SHTxx and the internal calibration data, the sensor can compensate for the humidity cross-sensitivity. The compensated signal, shown as solid lines in Figure 8, only contains minimal humidity cross-sensitivity over the entire range from 20% to 80% relative humidity.

### 3.4. CO_2_ equivalent 

Volatile organic compounds in indoor environments mainly originate from two sources: human activities and outgassing from furnishings and building materials. The human contribution to indoor air pollutants has been historically correlated with CO_2_ which is commonly used as an indicator for insufficient ventilation in closed spaces [51,52,53]. For this reason, the concept of an CO_2_ equivalent (CO2eq) has been suggested which is in fact a rescaled tVOC signal from a MOx sensor [54,55]. While a VOC-based CO2eq clearly captures more air-pollution events than a CO_2_ measurement, the rescaling of the tVOC signal into a CO2eq does not provide additional information when compared to a tVOC signal alone.

In contrast, a multi-pixel approach offers the possibility to increase or tune the selectivity by combining multiple signals with different response curves. As a first step in this direction, the SGP30 does offer a second raw signal which has an increased selectivity to hydrogen (H_2_). We propose that such a signal could be used to distinguish the influence of a human presence from other contaminants. The reason is that in indoor environments, the H_2_ concentration is expected to correlate well with the CO_2_ concentration as human breath contains significant concentrations of both, CO_2_ (4%) and H_2_ (10 ppm) [56]. Furthermore, humans are the only major source of CO_2_ and H_2_ in typical indoor environments.

**Figure 9:**
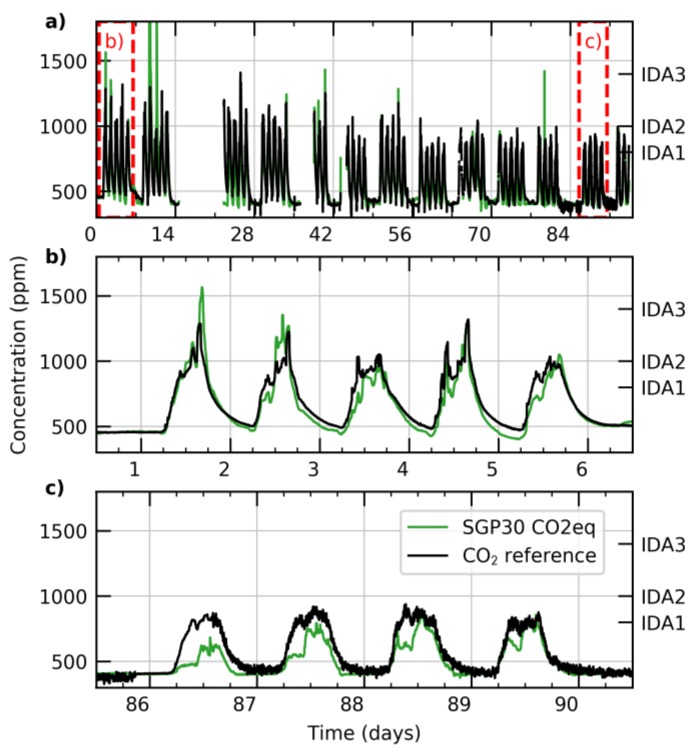
CO_2_ data taken over about 3 months in a typical corporate meeting room (**a**). The output of the non-dispersive infrared (NDIR) CO_2_ reference (black line) is compared to the output of the SGP CO2eq algorithm (green line). The two red rectangles indicate the position of the zoom-ins provided in panels (**b**,**c**). On the secondary y-axis the maximum values of the corresponding indoor air-quality (IDA) levels are indicated [53].

In the SGP, the raw H_2_ signal is baseline-corrected and then converted to an equivalent CO_2_ value in ppm, called CO_2_ equivalent (CO2eq). The conversion factor has been established by correlating the H_2_ signal to CO_2_ values measured with NDIR CO2 sensors in extensive field studies. Figure 9 presents CO2eq data from a SGP sensor in comparison with a conventional NDIR CO_2_ sensor signal. The data was taken in a meeting room in a regular office building over a span of more than 3 months. The graphs are colored to represent the indoor air-quality (IDA) levels as specified by the European standard on room ventilation [53]. Overall a good correlation between the optically measured CO_2_ value and the equivalent value can be found. Remaining deviations can be attributed to a limited cross-sensitivity to large tVOC signals and variations in individual H_2_ and CO_2_ breath concentrations. In addition to the tVOC signal, the CO2eq might be an attractive low-cost alternative to optical CO_2_ sensors, in particular for applications where a human presence is the major contribution to air quality (e.g., meeting rooms, schools).

## 4. Conclusions

The new SGP platform represents a milestone in the commercial application of MOx technology. The unique combination of sensor and electronics integration, multi-pixel platform and long-term stability opens a pathway to novel applications for metal-oxide gas sensors in consumer products like mobile phones, wearables and IoT devices.
